# Genome-Wide Identification of *bZIP* Gene Family in *Lycium barbarum* and Expression During Fruit Development

**DOI:** 10.3390/ijms26104665

**Published:** 2025-05-13

**Authors:** Han Gao, Xiaoyu Cao, Yunni Ma, Xiaoya Qin, Xiaorong Bai, Xiyan Zhang, Aisheng Xiong, Yue Yin, Rui Zheng

**Affiliations:** 1 Key Laboratory of Ministry of Education for Protection and Utilization of Special Biological Resources in Western China, College of Life Sciences, Ningxia University, Yinchuan 750021, China; hangao2000@163.com; 2 National Wolfberry Engineering Research Center, Ningxia Academy of Agriculture and Forestry Sciences, Yinchuan 751002, China; cxyu1025@nwafu.edu.cn (X.C.); qinxiaoya@whu.edu.cn (X.Q.); xiaorongb0206@163.com (X.B.); zhangxy911@163.com (X.Z.); 3 College of Food Science and Engineering, Ningxia University, Yinchuan 750021, China; yunnima0513@163.com; 4 State Key Laboratory of Crop Genetic and Germplasm Enhancement, College of Horticulture, Nanjing Agricultural University, Nanjing 210095, China; xiongaisheng@njau.edu.cn; 5 State Key Laboratory of Efficient Production of Forest Resources, Beijing 100091, China

**Keywords:** *Lycium barbarum*, *bZIP* gene family, evolutionary analysis, gene expression, subcellular localization

## Abstract

Wolfberry (*Lycium barbarum* L.) is a valued traditional medicinal plant and dietary supplement in China. The basic leucine zipper (bZIP) transcription factor (TF) family is a multifunctional group of regulatory proteins critical to plant biology, orchestrating processes such as growth and development, secondary metabolite biosynthesis, and stress responses to abiotic conditions. Despite its significance, limited information about this gene family in wolfberry is available. In this study, a total of 66 *LbabZIP* genes were identified, exhibiting a non-uniform distribution across all 12 chromosomes. Phylogenetic analysis divided these genes into 13 subgroups based on comparison with *Arabidopsis* bZIP proteins. Analysis of gene structures and conserved motifs revealed high similarities within individual subgroups. Gene duplication analysis indicated that dispersed duplication (DSD) and whole-genome duplication (WGD) events were the primary drivers of *LbabZIP* gene family expansion, with all duplicated genes subject to purifying selection. *Cis*-regulatory element (CRE) analysis of *LbabZIP* promoter regions identified numerous elements associated with plant growth and development, hormone signaling, and abiotic stress responses. Gene Ontology (GO) annotation further indicated that the *LbabZIP* genes are involved in transcriptional regulation, metabolism, and other biological processes. Transcriptome data and quantitative real-time PCR (qRT-PCR) analysis demonstrated tissue-specific expression patterns for several *LbabZIP* genes. Notably, *LbaZIP21/40/49/65* showed significant involvement in wolfberry fruit development. Subcellular localization assays confirmed that these four proteins are nucleus-localized. This comprehensive analysis provides a theoretical foundation for future studies investigating the biological functions of *LbabZIP* genes, especially their role in wolfberry fruit development.

## 1. Introduction

Wolfberry (*Lycium barbarum* L.), a perennial shrub of the Solanaceae family, is widely cultivated in Northwest China’s arid and semi-arid regions. As an important medicinal and edible plant, the fruits of *L. barbarum* are rich in polysaccharides, carotenoids, amino acids, and flavonoids, and it has been reported to have immune-enhancing, anti-aging, cancer prevention, and anti-oxidative properties [1,2]. The development and maturation of wolfberry fruit represent crucial phases in its growth and overall development. The process of fruit development is highly regulated, with a set of genes playing key roles in the development of wolfberry fruit [3]. Moreover, with the report of the wolfberry genome sequence [4], several transcription factor (TF) families, such as R2R3-MYB, BBX, GATA, and PYL, have been noted [5,6,7,8]. However, this is just an iceberg of the complex transcriptional network in wolfberry.

The basic leucine zipper (bZIP) TF family is one of the largest and most diverse families of TF in plants and other eukaryotes. Structurally, members of the bZIP TFs are characterized by a highly conserved basic region at the N-terminus and a leucine zipper region at the C-terminus. The basic region is highly conserved, comprising ~16 amino acids with an invariant N-X7-R/K-X9 motif that recognizes ACGT-core DNA sequences, including A-box (TACGTA), C-box (GACGTC), and G-box (CACGTG) [9,10]. To date, the bZIP TF family has been identified and analyzed in several plants, including 78 in Arabidopsis [9], 86 in rice (*Oryza sativa*) [11], 69 in tomato (*Solanum lycopersicum*) [12], 92 in pear (*Pyrus breschneideri*) [13], 49 in potato (*Solanum tuberosum*) [14], 61 in wax gourd (*Benincasa hispida*) [15], and 45 in jujube (*Ziziphus jujuba*) [16]. However, basic information of the bZIP TFs in wolfberry is still uncovered.

Previous research reported that the roles of bZIP families are varied in regulating plant development and secondary metabolism [17,18]. For example, heterologous expression of *FvbZIP11* in tomato significantly increased the fruit total soluble solid and soluble sugar content [19]. Overexpression of *PgbZIP16* and *PgbZIP34* from pomegranate plants in Arabidopsis promotes the accumulation of anthocyanin in Arabidopsis leaves [20]. In plum, PsbZIP1 and PsbZIP10 promoted anthocyanin synthesis by binding directly to the G-box of the *PsUFGT* promoter [21]. And citrus CsbZIP44 directly binds to the promoters of four carotenoid metabolism-related genes (*CsDXR*, *CsGGPP*s, *CsBCH1*, and *CsNCED2*) and activates their expression [22]. In addition, SlHY5 and SlPIF3 were involved in the biosynthesis of steroidal glycoalkaloids mediated by light signals by controlling the expression of *SlGAME1*, *SlGAME4*, and *SlGAME17* in tomato leaves [23]. Therefore, the bZIP family of proteins is involved in and plays an important role in secondary metabolite synthesis during fruit development, such as for flavonoids, carotenoids, and alkaloids.

In this study, the potential role of *LbabZIPs* in regulating plant growth and development, particularly in wolfberry fruit development, was investigated. A total of 66 *LbabZIP* genes were identified and classified in the wolfberry genome using bioinformatics approaches. The gene structure, motif composition, and conserved domains were analyzed to identify the conservation and specificity of *LbabZIPs*. Furthermore, a comparative analysis of *bZIP* genes from wolfberry, tomato, pepper, Arabidopsis, and rice was performed to predict the functions of *LbabZIP* genes. The roles of specific *LbabZIP* genes in various biological processes were characterized through expression pattern analysis across different wolfberry tissues. More importantly, the expression of *LbabZIP* genes during fruit development comprehensively analyzed by RT-qPCR indicated that some *LbabZIP* genes were responsive to fruit development. This study provides valuable information for identifying candidate *LbabZIP* genes involved in regulating the growth and development of wolfberry, particularly fruit development.

## 2. Results

### 2.1. Identification and Characteristics of bZIP Genes in Wolfberry

To systematically identify bZIP TFs in the wolfberry genome, a homology-based BLASTP search was conducted using 78 experimentally validated *Arabidopsis thaliana* bZIP protein sequences as queries. The candidate sequences were confirmed to contain the bZIP domain using the Simple Modular Architecture Research Tool (SMART). A total of 66 *LbabZIP* genes were systematically identified in the *Lycium barbarum* genome and sequentially designated *LbabZIP1* to *LbabZIP66* according to their chromosomal locations (Supplementary Table S1). The amino acid lengths of the encoded LbabZIP proteins ranged from 129 amino acids (LbabZIP16) to 813 amino acids (LbabZIP31 and LbabZIP46), with molecular weight (MW) ranging from 14.98 kDa (LbabZIP16) to 92.17 kDa (LbabZIP31). The isoelectric points (pI) varied from 4.97 (LbabZIP37) and 9.93 (LbabZIP6*2*). All LbabZIP proteins had negative grand average of hydropathicity (GRAVY) values, indicating their hydrophilic nature. Most of the LbabZIP proteins were unstable, except LbabZIP35 and LbabZIP31. Subcellular localization prediction revealed that the majority of LbabZIP proteins in *Lycium barbarum* are predominantly localized to the nucleus, consistent with their functional role as transcription factors regulating gene expression.

### 2.2. Phylogenetic Relationship and Classification of LbabZIP Proteins

**Figure 1:**
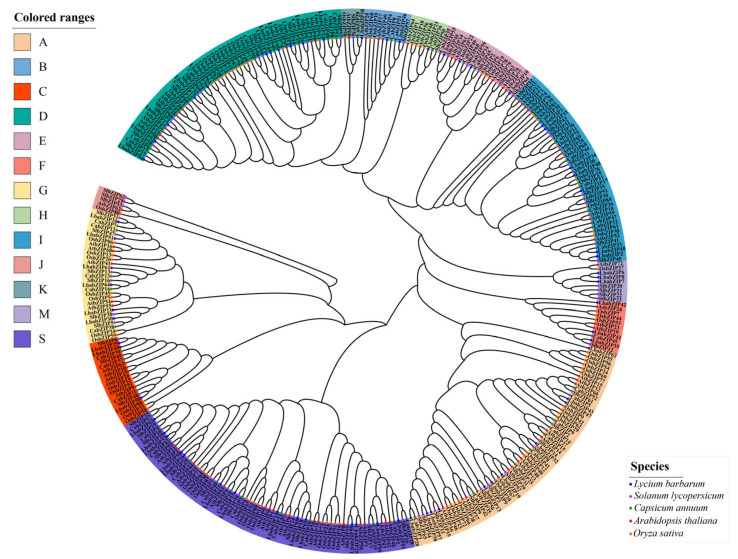
Phylogenetic analysis of *bZIP* genes from wolfberry, tomato, pepper, *Arabidopsis*, and rice. Wolfberry bZIP proteins are divided into 13 distinct clades. Protein sequence alignment was performed using MUSCLE, and a neighbor-joining phylogenetic tree was reconstructed with 10,000 bootstrap replicates. Colored regions represent different subgroups. Wolfberry bZIP proteins (blue solid circles), tomato bZIP proteins (purple solid circles), pepper bZIP proteins (green solid circles), Arabidopsis bZIP proteins (red solid circles), and rice bZIP proteins (orange solid circles) are denoted.

To explore the evolutionary relationships and classification of the *LbabZIP* family, a phylogenetic tree was constructed using 353 bZIP protein sequences from *L. barbarum* (66), *S. lycopersicum* (69)*, C. annuum* (54)*, O. sativa* (86), and *A. thaliana* (78) (Figure 1). Based on their relationship with *A*. *thaliana bZIP* genes, the *LbabZIP* genes were divided into 13 subgroups: A, B, C, D, E, F, G, H, I, J, K, M, and S. Subgroup S contained the largest number of *LbabZIP* genes (14), followed by subgroup D (12) and subgroup A (11). Subgroups K and H contained only one gene each. Notably, no *LbaZIP* member was assigned to subgroup J.

### 2.3. Analysis of Gene Structure and Conserved Motifs in LbabZIP Genes

**Figure 2:**
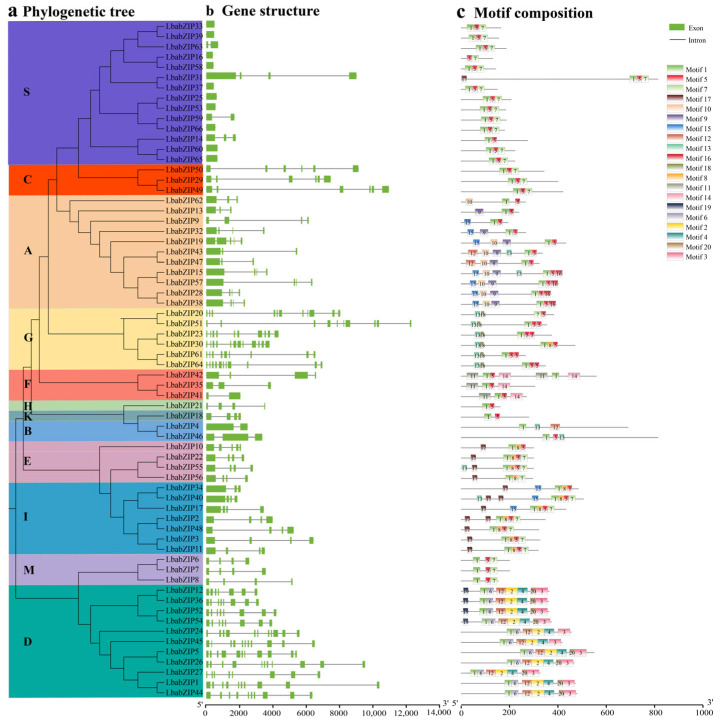
Phylogenetic relationships, gene structure, and motif distributions of wolfberry *bZIP* genes. (**a**) The phylogenetic tree was generated from full-length sequences using MEGA 6.06. (**b**) The exon–intron structure of wolfberry *bZIP* genes, with yellow boxes indicating exons and gray lines indicating introns. (**c**) Conserved motif distributions in wolfberry proteins. Motifs 1–20 are represented by colored boxes. Motif sequence details are available in Supplementary Table S2. The scale bar indicates protein length.

The gene structure of *LbabZIP* genes was analyzed by examining their exon–intron structure (Figure 2b). Gene structure analysis of the *LbabZIP* genes in *Lycium barbarum* revealed significant diversity in intron numbers, ranging from 0 to 11 introns per gene, with a mean of 3.9 introns. Subgroup-specific patterns were observed; for example, most genes in subgroup S slacked introns, while subgroup G had the largest number of introns.

The conserved motifs of LbabZIP proteins were identified using the MEME analysis, revealing 20 motifs (Figure 2c and Supplementary Table S2). Motif 1 was conserved in all LbabZIP proteins except LbabZIP20. Motifs 1, 5, and 7 were commonly detected across most subgroups, whereas subgroup-specific motifs included motif 13 and motif 18 (subgroup G), motif 10 (subgroup A), and motifs 2, 3, 4, and 6 (subgroup D). Motif 14 was unique to three members in subgroup F. These conserved motifs may reflect specific biological functions.

### 2.4. Chromosomal Distribution of bZIP Genes in Wolfberry

**Figure 3:**
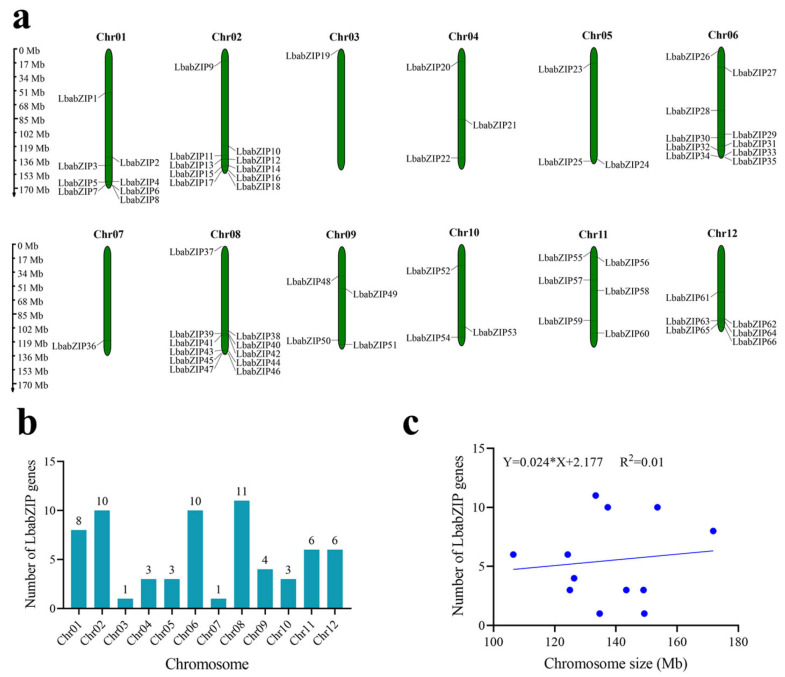
The chromosomal localization of wolfberry *bZIP* genes. (**a**) The distribution of *LbabZIP* genes across 12 chromosomes, with chromosome numbers labeled at the top and scales in megabases (Mb). (**b**) The number of *LbabZIP* genes on each chromosome. (**c**) The correlation between chromosome size and the number of *LbabZIP* genes.

The 66 *LbabZIP* genes were unevenly distributed across 12 wolfberry chromosomes (Figure 3a). Chromosome 8 (Chr8) contained the largest number of *LbabZIP* genes (11, 16.6%), followed by Chr02 and 6, each with 10 genes (15.1%). Chromosomes 3 and 7 contained only one gene each (*LbabZIP19* and *LbabZIP36*, respectively, Figure 3b). All chromosomes contained at least one *bZIP* gene. Interestingly, despite being the longest chromosome, chromosome 1 contained only eight *bZIP* genes, indicating no clear correlation between chromosome size and gene number (Figure 3c).

### 2.5. Synteny, Duplication Events, and Selective Pressure Analysis of LbabZIP Genes

**Figure 4:**
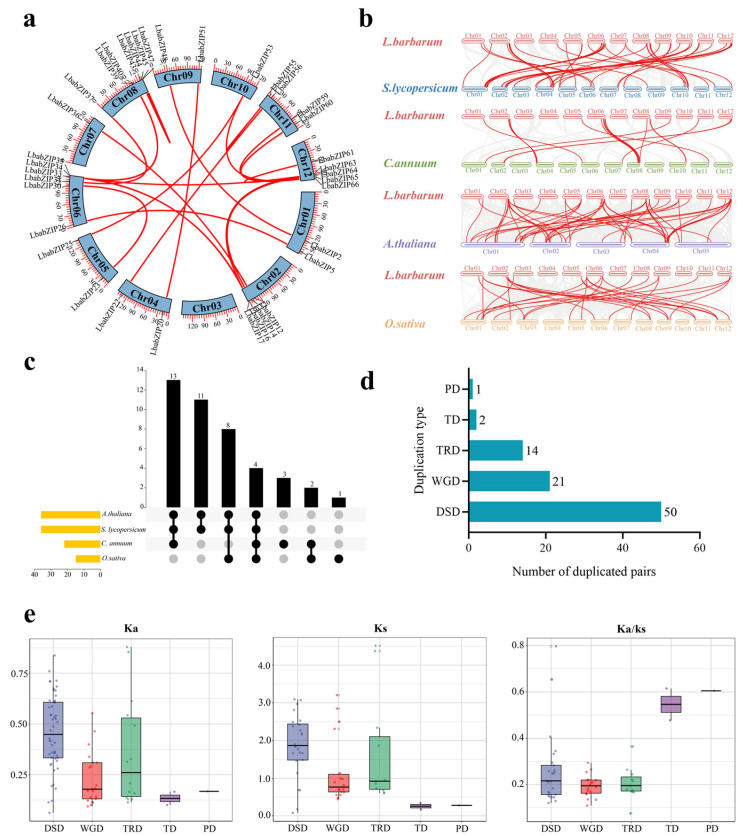
Synteny, duplication events, and selective pressure analysis of wolfberry *bZIP* genes. (**a**) Synteny analysis of *LbabZIP* genes within wolfberry genome. Red lines highlight syntenic *bZIP* gene pairs. (**b**) Synteny analysis of *bZIP* genes between wolfberry and tomato, pepper, Arabidopsis, and rice genomes, with red lines indicating syntenic *LbabZIP* gene pairs. (**c**) Syntenic *bZIP* gene pairs across wolfberry and four other species are represented by vertically connected blocks and yellow columns. (**d**) Distribution of duplicated *LbabZIP* genes across five duplication types: transposed duplication (TRD), whole-genome duplication (WGD), dispersed duplication (DSD), tandem duplication (TD), and proximal duplication (PD). (**e**) Ka, Ks, and Ka/Ks values for duplicated *LbabZIP* gene pairs from five duplication events.

Intra-species synteny analysis revealed 22 paralogous *LbabZIP* gene pairs, residing in 19 syntenic blocks. The smallest block contained 8 gene pairs and had 1 *LbabZIP* gene pair, while the largest block comprised 81 gene pairs and contained 1 *LbabZIP* gene pair. Comparative syntenic maps were plotted between *L. barbarum* and four plant species, including *S. lycopersicum*, *C. annuum*, *A. thaliana*, and *O. sativa*. These analyses revealed 53, 22, 55, and 27 homologous *bZIP* gene pairs, respectively (Figure 4b and Supplementary Table S3), suggesting a closer evolutionary relationship between *L. barbarum* and *S. lycopersicum*/*A. thaliana*. Further analysis revealed that 4 *bZIP* syntenic gene pairs were shared by all four plant species and 11 *bZIP* syntenic gene pairs were shared between two Solanaceae species (tomato and pepper) (Figure 4c).

To investigate the expansion and evolution of the *LbabZIP* gene family, five duplication types (WGD—whole-genome duplication, DSD—dispersed duplication, TRD—transposed duplication, TD—tandem duplication, and PD—proximal duplication) were identified. A total of 86 duplication events were observed, with DSDs contributing the highest number (50 pairs), followed by WGD (21 pairs), TRD (14 pairs), TD (2 pairs), and PD (1 pair) (Figure 4d). These results suggest that dispersed duplication played a predominant role in the evolution of the *LbabZIP* gene family.

Selective pressure analysis showed that the *Ka/Ks* ratios for all duplicated gene pairs were less than 1, indicating that *LbabZIP* genes underwent purifying selection during evolution (Figure 4e and Supplementary Table S4).

### 2.6. Cis-Element Analysis of the LbabZIP Genes

**Figure 5:**
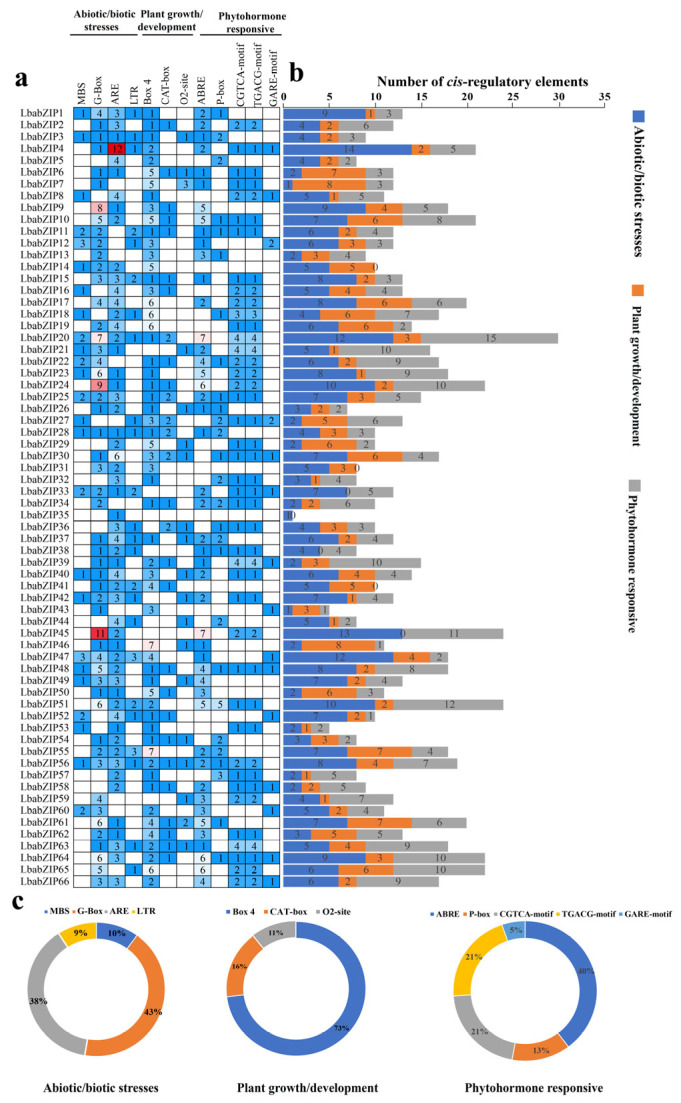
*Cis*-regulatory elements analysis of *LbabZIP* gene promoters. (**a**) Number and type of *cis*-regulatory elements in *LbabZIP* genes, numbers, and colors indicating different elements. (**b**) Categorization of *cis*-regulatory elements of *LbabZIP* genes into three subgroups (biotic and abiotic stresses, plant hormones, and plant growth). (**c**) Pie charts showing the relative proportions of *cis*-regulatory elements in each subgroup.

A total of 896 *cis*-regulatory elements were identified and divided into three main subgroups: abiotic and biotic stress responses, plant growth and development, and phytohormone responsiveness (Figure 5 and Supplementary Table S5). In the abiotic and biotic stress subgroup, four types of *cis*-elements were identified, including MBS (involved in drought inducibility), G-Box (plant growth response to light), ARE (responsible for anaerobic induction), and LTR (low-temperature response). Among these, G-Boxes was the most prevalent *cis*-element, accounting for 43% of the total. In the plant growth and development subgroup, the identified cis-elements included Box 4, CAT-box, and O2-site, which are associated with light-induced growth, meristem-related processes, and zein metabolism, respectively. Box 4 accounted for the highest proportion (73%), suggesting a significant role for LbabZIP genes in light-regulated growth responses. In the phytohormone-responsive subgroup, five cis-elements were identified: ABRE (abscisic acid response), P-box, GARE-motif (gibberellin response), and CGTCA-motif and TGACG-motif (jasmonic acid methyl ester response). Notably, the *LbabZIP* genes contained 129 ABRE elements (40%), with the CGTCA-motif and TGACG-motif collectively accounting for 68%. These findings suggest that *LbabZIP* genes are involved in plant growth and development, hormone response, and stress adaptation.

### 2.7. Gene Enrichment Analysis of the LbabZIP Genes

**Figure 6:**
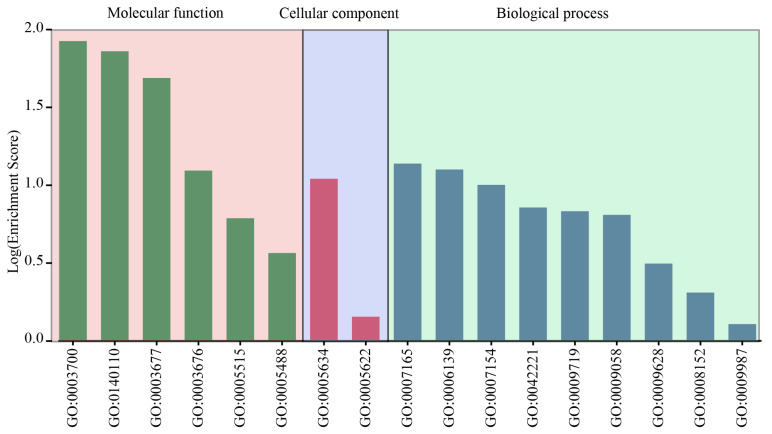
GO enrichment analysis of wolfberry *bZIP* genes. Gene Ontology (GO) enrichment analysis categorized the *LbabZIP* genes into cellular components, biological processes, and molecular functions.

To elucidate the functional classification of *LbabZIP* genes, the egg-NOG-mapper online tool was employed for Gene Ontology (GO) analysis. The predicted GO terms were categorized into three main groups: biological process, cellular component, and molecular function, distributed across 17 distinct pathways (Figure 6 and Supplementary Table S6). Among these pathways, nine were associated with biological processes, primarily signal transduction, nucleobase-containing compound metabolism, and cell communication. Molecular function-related pathways included DNA-binding transcription factor activity, transcription regulator activity, and DNA binding. The cellular component group had two pathways, reflecting the roles of LbabZIP proteins in specific cellular locations.

### 2.8. Expression Patterns of bZIP Genes in Different Tissues of Wolfberry

**Figure 7:**
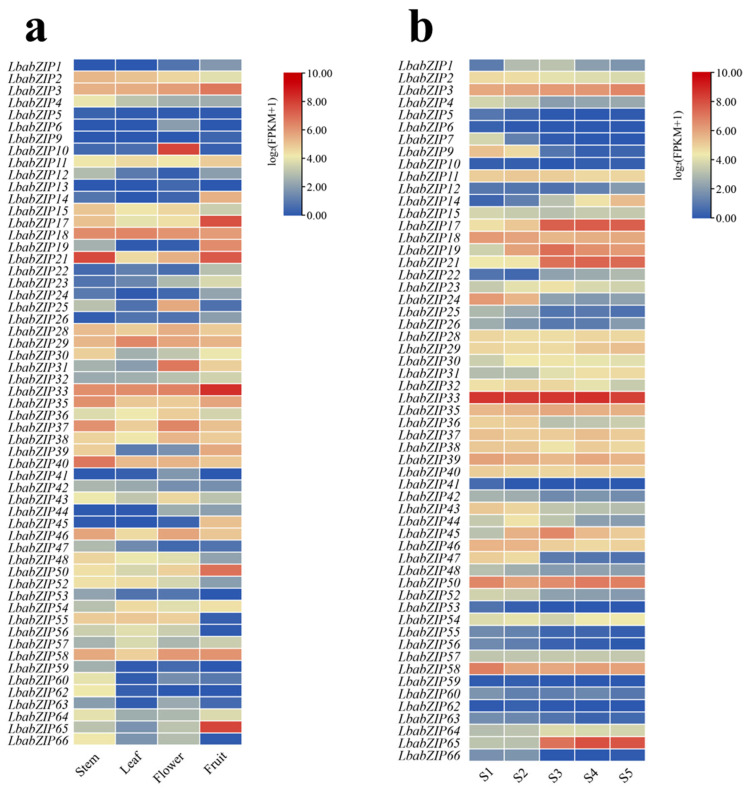
Expression profiles of *LbabZIP* genes in wolfberry tissues and fruits. (**a**) Heatmap representing *LbabZIP* gene expression in four wolfberry tissues. (**b**) Heatmap of *LbabZIP* gene expression during five fruit developmental stages of *Lycium barbarum* var. *auranticarpum*. S1, S2, S3, S4, and S5 periods represent 12, 19, 25, 30, and 37 days after full bloom (DAF), respectively. FPKM values were Log_2_-transformed (value + 1), with red and blue indicating high and low expression levels, respectively.

To explore roles of *bZIP* genes, we conducted a comprehensive expression analysis utilizing transcriptome sequencing data from multiple wolfberry tissues (stems, leaves, flowers, and fruits) and five distinct fruit developmental stages. Of the 66 *LbabZIP* genes, eight were not expressed in any tissue (Figure 7a and Supplementary Table S7), and nine showed no expression during fruit development (Figure 7b and Supplementary Table S8). Tissue-specific expression revealed diverse roles for *LbabZIP* genes. For example, *LbabZIP4*, *LbabZIP12*, *LbabZIP40*, and *LbabZIP47* were highly expressed in stems, while *LbabZIP18*, *LbabZIP54*, and *LbabZIP55* were highly expressed in flowers, indicating potential roles in flower development. Nine genes (*LbabZIP3/9/17/19/21/24/33/50/65*) exhibited high expression in fruits. Notably, the expression of *LbabZIP17*, *LbabZIP21,* and *LbabZIP65* increased progressively during fruit development, whereas *LbabZIP9*, *LbabZIP24*, and *LbabZIP47* showed a declining trend (Figure 7b). These results suggest that *bZIP* genes play crucial roles in fruit development.

### 2.9. Expression Validation by qRT-PCR

**Figure 8:**
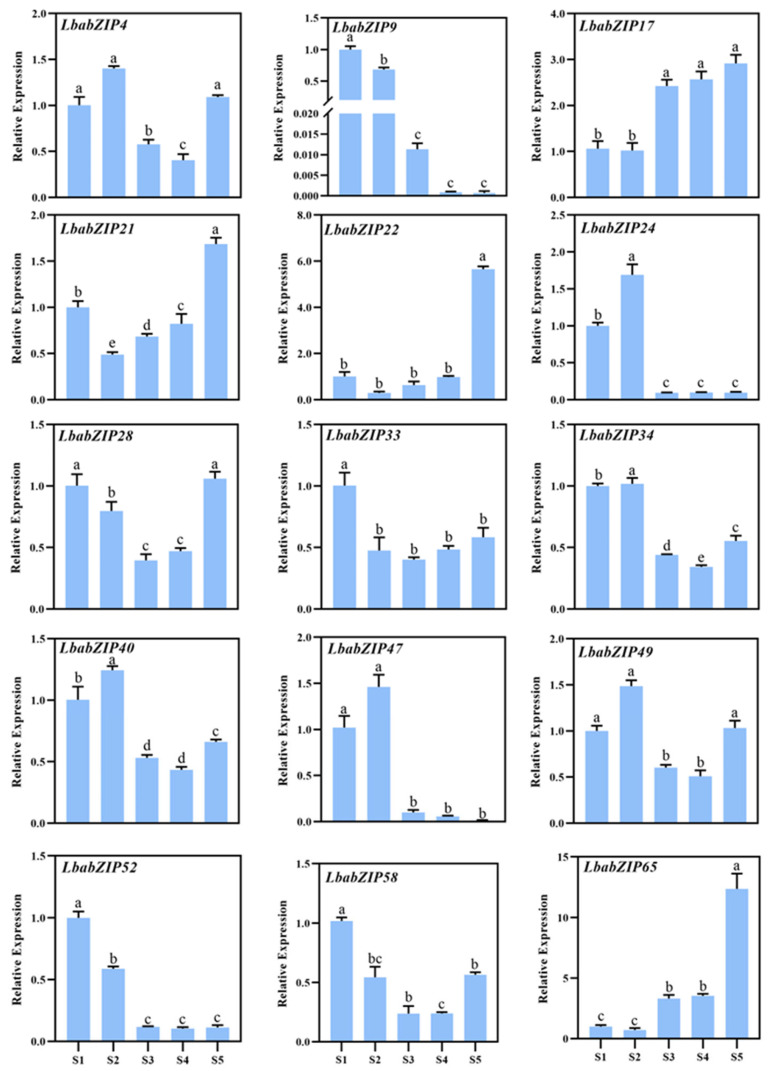
Expression patterns of 15 *LbabZIP* gene determined by qRT-PCR. Expression levels were normalized to the wolfberry *LbaActin* gene and analyzed using the 2^−ΔΔCt^ method. S1, S2, S3, S4, and S5 period represent 12, 19, 25, 30, and 37 days after full bloom (DAF), respectively. Data are presented as the mean ± standard deviation (SD). Different letters indicate significant differences (*p* < 0.05, n = 3).

To validate transcriptome results, the expression of 15 representative *LbabZIP* genes was analyzed at five stages of wolfberry fruit development using qRT-PCR (Figure 8 and Supplementary Table S9). Consistent with transcriptome data, the expression levels of *LbabZIP17, LbabZIP21, LbabZIP22*, and *LbabZIP65* gradually increased and peaked at the S5 stage. In contrast, *LbabZIP9, LbabZIP24, LbabZIP47,* and *LbabZIP52* exhibited decreasing expression patterns. This consistency suggests that *LbabZIP* genes play a regulatory role in fruit development.

### 2.10. Subcellular Localization of LbabZIP Proteins

**Figure 9:**
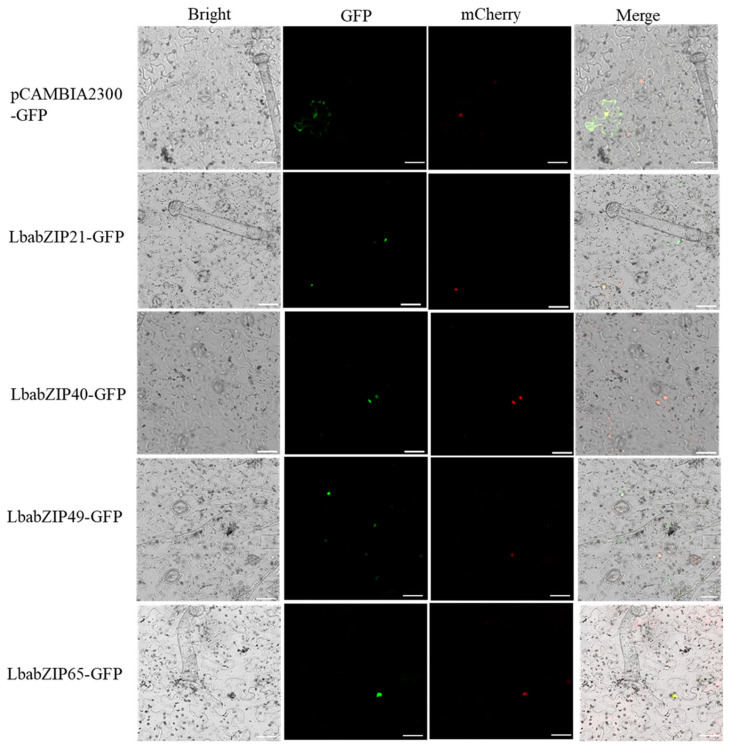
Subcellular localization of *LbabZIPs* proteins. *LbabZIPs*-GFP fusion proteins were transiently expressed in *N. benthamiana* leaves and visualized via fluorescence microscopy 48 h post-infiltration. The 35S-GFP was used as positive control. From left to right: bright field; green fluorescent protein (GFP); mCherry is a red fluorescent protein (RFP) fused with the nucleus marker; Merge indicates merger between GFP and mCherry fluorescence images. Scale bars = 20 μm.

To determine the subcellular localization of LbabZIP21/40/49/65 proteins, GFP-tagged fusion proteins were transiently expressed in *Nicotiana benthamiana* leaf epidermal cells. Fluorescence microscopy revealed that GFP signals for the control were ubiquitously distributed throughout the cells, while the fusion proteins (LbabZIP21/40/49/65) were confined in the nucleus (Figure 9). These results confirm that *LbabZIP21*, *LbabZIP40*, *LbabZIP49*, and *LbabZIP65* encode nuclear-localized proteins.

**Figure 10:**
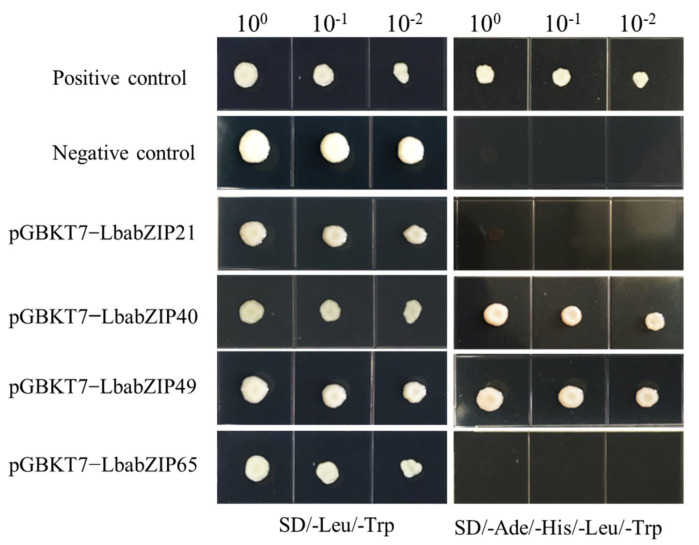
Transactivation activity of *LbabZIP* genes. *LbabZIP21*, *LbabZIP40*, *LbabZIP49*, and *LbabZIP65* coding sequences were cloned into the pGBKT7 vector and transformed into yeast strain AH109. Yeast cells were dotted at 10^−1^ dilution on SD/-Leu/-Trp and SD/-Ade/-His/-Leu/-Trp media. Positive control: pGBKT7-53 + PGADT7-RecT; negative control: pBGKT7 vector alone.

To evaluate the transcriptional activity of four *LbabZIP* TFs (*LbabZIP21, LbabZIP40, LbabZIP49,* and *LbabZIP65*) in vivo, a yeast two-hybrid (Y2H) assay was conducted using the pGBKT7-*LbabZIP* constructs expressed in *Saccharomyces cerevisiae* strain AH109. Yeast cells harboring the pGBKT7-*LbabZIP40* and pGBKT7-*LbabZIP49* constructs and the positive control (pGBKT7-53 + PGADT7-RecT) grew successfully on the selective medium SD/-Ade/-His/-Leu/-Trp. In contrast, yeast cells containing pGBKT7-*LbabZIP21* and pGBKT7-*LbabZIP65* and the negative control (empty vector pGBKT7) failed to grow in the same medium (Figure 10). These findings demonstrate that *LbabZIP40* and *LbabZIP49* have transactivation activities in the yeast assay system, whereas *LbabZIP21* and *LbabZIP65* do not exhibit such activity under these experimental conditions.

## 3. Discussion

bZIP TFs is one of the largest transcription factor families that widely exist in eukaryotes and they have been highly conserved in evolution [9]. bZIP TFs play crucial roles in regulating plant growth and development, secondary metabolism, and responses to abiotic stress [17,18,24]. The *bZIP* gene family has been extensively studied in many plants, with 78 members identified in *Arabidopsis* [9], 86 in *Oryza sativa* [11], 69 in *Solanum lycopersicum* [12], 52 in *Capsicum annuum* [25], 62 in *Pyrus bretschneideri* [13], 59 in *Castanea mollissima* [26], 92 in *Hordeum vulgare* [27], and 45 in *Liriodendron chinensis* [28]. However, until now, although the entire wolfberry genome has been sequenced [4], studies of *bZIP* gene family in wolfberry are limited. In this study, we performed a genome-wide analysis of wolfberry and identified a total of 66 *LbabZIP* genes. It is similar to the number of *bZIP* gene family members in *Solanum lycopersicum* (66) [12], *Pyrus bretschneideri* (62) [13], and of other species. The *bZIP* genes in wolfberry were classified into 13 subgroups by constructing phylogenetic trees of the *bZIP* gene family in *Lycium barbarum*, *Solanum lycopersicum*, *Capsicum annuum*, *Oryza sativa*, and *Arabidopsis*, which is similar to the grouping of the *Arabidopsis bZIP* gene family [9] (Figure 2). In addition to the absence of *bZIP* gene family members of *L. barbarum* in group J, the *bZIP* gene numbers of five species were distributed in other subgroups. The suggests that the number of *bZIP* genes is more conserved during evolution. Due to the highly conserved nature of bZIP sequences, genes within the same *bZIP* group exhibit identical or similar functions. This conservation provides a valuable reference for investigating the functional roles of this gene family [29].

The analysis of gene structure and conserved motifs can facilitate the prediction of gene functions [15]. The results of structural analysis of wolfberry *bZIP* genes revealed that the *bZIP* gene structure of wolfberry is relatively simple, with the number of introns ranging from 0 to 11. It can be divided into two categories according to the presence or absence of introns. A total of 10 *bZIP* genes without introns was found in the genome of wolfberry. All 10 *bZIP* genes were classified in group S. A similar situation exists in species such as *Arabidopsis*, *Nelumbo*, and *Suaeda australis* [9,30,31]. The *bZIP* genes categorized into various subgroups may serve distinct functions in plant growth and development. For example, most of members of group A are involved in abiotic stress responses, such as drought, salinity, and cold stress. Genes located in group C are associated with seed maturation and storage protein synthesis [32]. Genes located in group S are often linked to sugar signaling and metabolism [33]. Genes located in group G are implicated in light signaling and photomorphogenesis [34]. The number of conserved motifs present in each bZIP protein also exhibits significant variability. Some bZIP proteins contain only two conserved motifs; most proteins have three conserved motifs (Figure 2). In addition to the specific bZIP_1 (PF00170) and bZIP_2 (PF07716) domains, plant bZIP proteins also possess additional functionally conserved domains that are involved in various biological processes. In wolfberry, the identified bZIP members contain three distinct conserved domains: BRLZ, bZIP_C, and bZIP_2. Among them, the BRLZ conserved domain is widely present in the *bZIP* gene family of wolfberry. bZIP_C is found in two members (*LbabZIP29* and *LbabZIP49*), while bZIP_2 is only present in *LbabZIP31* (Figure 2).

Previous studies have found that the expansion and contraction of gene families are mainly driven by gene duplication, such as whole-genome duplication, dispersed duplication, and tandem duplication [35,36]. For example, the expansion of *BBX* and *LBD* gene families was primarily driven by whole-genome duplication and dispersed duplication [35,37]. In this study, gene duplication analysis revealed that dispersed duplication and whole-genome duplication events drove the expansion of the *LbabZIP* gene family. Fifty *LbabZIP* genes (58.13%) were grouped into the DSD type, and 21 genes (24.41%) belonged to the WGD type, which might be due to the high ratio of self-incompatibility and the domestication process of wolfberry [4]. Following gene duplication, duplicated gene pairs can experience three main different types of selection pressures, including positive selection, negative selection (also known as purifying selection), and neutral selection [37]. Based on the estimated results of *Ka*/*Ks*, our analysis revealed that the evolution of the *LbabZIP* gene family was mainly driven by purifying selection.

Gene transcription is significantly impacted by the *cis*-acting element of the promoter, which can directly alter gene expression and function [38]. The function of the gene can be predicted or inferred based on the types of *cis*-acting element present in its promoter region. Among the promoters of the 66 *LbabZIP* gene family members in wolfberry, most of *cis*-elements were related to stresses, plant growth, and development and phytohormones. Previous studies have been verified that show that the *bZIP* genes are regulated by hormones such as ABA and IAA in the regulation of plant growth and development [39,40,41]. For instance, *OsABF2* in rice binds to ABREs to regulate abiotic stress-responsive genes via an ABA-dependent pathway [42], and *PpABF3* targets the G-box element in the *PpWRKY44* promoter, enhancing salinity-induced malate accumulation [43]. In apples, *MdbZIP44* enhances anthocyanin accumulation in response to ABA by facilitating *MdMYB1* binding to target gene promoters [41]. Many hormone-responsive elements were also detected in the *LbabZIP* promoter region: ABRE, P-box, CGTA-motif, TGACG-motif, and GARE-motif. Therefore, the *LbabZIP* gene family plays a crucial role in plant growth, development, and stress resistance in wolfberry by mediating plant hormone regulation.

Increasing evidence suggests that *bZIP* genes have varied functions during plant development processes. Generally, patterns of gene expression can offer significant insights for predicting gene function [37]. To investigate *LbabZIP* gene expression patters, transcriptome sequencing and qRT-PCR expression profiling were performed across four tissues and five fruit developmental stages. The *bZIP* gene family showed diverse expression patterns in four distinct tissues. This result showed that *LbabZIP* genes could be involved in a range of biological processes. Forty-seven genes are expressed throughout all tissues, indicating their extensive role in wolfberry growth and development. In addition, 12.12% of *LbaZIPs* were observed to have high transcriptional levels in wolfberry fruit, suggesting these genes may have important roles in development. Furthermore, qRT-PCR was used to confirm the expression levels of 15 randomly selected members of the *LbabZIP* gene family. Interestingly, we found that the S subgroup gene *LbabZIP65* was highly expressed in mature fruit, and is orthologous with *AtbZIP6* (*AT2G22850*) and *OsbZIP16* (*Os02g0191600*). Therefore, we speculated that the *LbabZIP65* gene might be involved in the regulation of fruit ripening and related color changes. However, the function of *bZIP* genes in fruits is still unknown and needs to be investigated further in future studies. Future research should employ techniques such as CRISPR/Cas9-mediated gene editing and RNA interference (RNAi) to directly manipulate *LbabZIP* gene expression in wolfberry. Investigation at the protein level, including protein–protein interaction studies and post-translational modification analyses, is needed to fully understand the regulatory mechanisms of *bZIP* TFs in wolfberry.

In this study, the *LbabZIP* gene family was identified, and its expression during fruit development was analyzed. Genes associated with fruit development, such as *LbabZIP65*, were excavated, which provided opportunities for manipulating fruit quality traits. Breeding strategies can be designed to regulate the expression of these genes, potentially leading to improvements in the fruit ripening process, the enhancement of coloration, and an increase in nutritional content. This provides important targets for the genetic improvement of wolfberry and holds significant importance for its breeding and production.

## 4. Materials and Methods

### 4.1. Plant Materials

*Lycium barbarum* var. *auranticarpum* plants were cultivated at the National Wolfberry Engineering Research Center, Yinchuan, Ningxia, China (37°53′ N, 105°72′ E). The fruit samples were collected on days 12 (S1), 19 (S2), 25 (S3), 30 (S4), and 37 (S5) after full-bloom (DAF), respectively. The fruit is yellow in color and nearly round in nature. The samples were immediately frozen in liquid nitrogen, and stored at −80 °C until further analysis.

### 4.2. Identification of bZIP Genes in the Wolfberry Genome

The completed *L. barbarum* genome sequence (Accession: GCA_019175385.2) was retrieved from the NCBI database (https://www.ncbi.nlm.nih.gov/datasets/genome/?taxon=112863, accessed on 15 January 2024) [4]. Two complementary methods were employed to identify *bZIP* genes in the wolfberry genome, as described in previous studies [6]. Initially, protein sequences of all *Arabidopsis* bZIP proteins, obtained from the TAIR database (https://www.arabidopsis.org/, accessed on 8 February 2024), were utilized as queries to perform a BLASTP search against the wolfberry genome database (E-value < 1 × 10^−5^). Secondly, the Hidden Markov Model (HMM) profile for the bZIP domain (PF00170) was downloaded from the Pfam database (http://pfam-legacy.xfam.org/, accessed on 15 January 2024) and used to screen the wolfberry protein database, with a threshold of an E-value < 1 × 10^−5^. Subsequently, candidate *LbabZIP* genes were validated by confirming the presence of the bZIP domain using SMART (http://smart.embl.de/, accessed on 15 January 2024) and the NCBI Conserved Domain Database (https://www.ncbi.nlm.nih.gov/cdd/, accessed on 15 January 2024). Protein properties, including molecular weight (Mw), isoelectric point (pI), and grand average of hydropathicity (GRAVY), were calculated using the Expasy online tool (https://web.expasy.org/compute_pi/, accessed on 16 January 2024). Subcellular localization predictions for *LbabZIP* proteins were performed using WoLF PSORT (https://wolfpsort.hgc.jp/, accessed on 16 January 2024) [44].

### 4.3. Phylogenetic Analysis of LbabZIP Proteins

To explore the evolutionary relationships of the wolfberry *bZIP* gene family, protein sequences of *Arabidopsis bZIP* genes (*AtbZIP*) were obtained from the TAIR database (https://www.arabidopsis.org/, accessed on 25 January 2024). Sequences of *bZIP* genes from two Solanaceae species (*Solanum lycopersicum* and *Capsicum annuum*) were retrieved from the SGN database (https://solgenomics.sgn.cornell.edu/, accessed on 25 January 2024). *O. sativa bZIP* genes were identified from previous studies [11]. All sequences were aligned using Muscle v5 [45], and a phylogenetic tree was constructed using the neighbor-joining method in MEGA6.06 [46] 1000 bootstrap replicates. Classification of the *LbabZIP* family was based on Arabidopsis bZIP classification criteria [9]. The phylogenetic tree was visualized using the iTOL platform (https://itol.embl.de/, accessed on 25 January 2024) [47].

### 4.4. Gene Structure and Conserved Motif Analysis of LbabZIP Genes

Exon–intron structures of *LbabZIP* genes were visualized using the Gene Structure Display Server 2.0 (GSDS) (https://gsds.gao-lab.org/Gsds_help.php, accessed on 20 January 2024) [48]. Conserved motifs in LbabZIP proteins were identified using the MEME suite (https://meme-suite.org/meme/, accessed on 20 January 2024), with the maximum number of motifs set to 20 [49].

### 4.5. Chromosomal Localization of LbabZIP Genes

Physical mapping of *LbabZIPs* genes was mapped to wolfberry chromosomes using MG2C v2.1 [50] based on genome location information.

### 4.6. Synteny, Duplication Events, and Selective Pressure Analysis of LbabZIP Gene Family

Collinearity analysis of *LbabZIP* genes was performed using TBtools-II [51]. Homology analysis between *L. bararum* and four other plant species (*S. lycopersicum*, *C. annuum*, *A. thaliana*, and *O. sativa*) was conducted using the dual synteny plotter in TBtools-II. Gene duplication events were identified using the Gen_finder tool (https://github.com/qiao-xin/DupGen_finder, accessed on 20 January 2024). The Ka, Ks, and Ka/Ks ratios were calculated using the Simple Ka/Ks Calculator (NG) model in TBtools v2.225 [51].

### 4.7. Cis-Element Analysis for LbabZIP Genes

To investigate regulatory elements associated with *LbabZIP* gene expression, promoter sequences (2000 bp sequence upstream of the start codon) were extracted using TBtools-II [52]. Cis-elements were identified using the PlantCARE database (http://bioinformatics.psb.ugent.be/webtools/PlantCARE/html/, accessed on 25 March 2024), and the results were visualized with TBtools v2.225 [51].

### 4.8. GO Analysis of the LbaZIP Genes

Gene Ontology (GO) term prediction for the *LbabZIP* genes was performed using the EggNOG-mapper tool (http://eggnog-mapper.embl.de/, accessed on 25 June 2024). The identified GO terms were categorized into three major categories: cellular components, biological processes, and molecular functions.

### 4.9. Expression Profiles of LbabZIP Genes Determined from RNA-Seq Datasets

Transcriptome sequencing (RNA-seq) data from five distinct developmental stages and four tissues of *Lycium barbarum* var. auranticarpum were retrieved from the NCBI database (accession number: PRJNA845109). The FPKM (fragments per kilobase of transcript per million mapped reads) values of *LbabZIP* genes were normalized through K-means clustering analysis using R software (version 3.2.2) [53]. Subsequently, the expression patterns were visualized using TBtools v2.225 software [51].

### 4.10. Total RNA Extraction and Expression Analysis of LbabZIP Genes

Primers specific to the *LbabZIP* genes were designed using Primer Premier 5 (Supplemental Table S9). Total RNA was extracted from wolfberry fruit of different stages using the E.Z.N.A.^®^Plant RNA Kit (OMEGA, Guangzhou, China). The first-strand cDNA was synthesized using the EasyScript^®^ One-Step gDNA Removal and cDNA Synthesis SuperMix Kit (TransGen, Beijing, China). qRT-PCR was performed using the PerfextStart^TM^ Green qPCR Super Mix (TransGen, Beijing, China) following the manufacturer’s instructions. The wolfberry *LbaActin* gene was used as the internal control for the normalization of gene expression. Relative expression levels were calculated using the 2^−ΔΔCt^ method [53]. Three independent biological replicates were used in the analysis. Statistical analyses were conducted using one-way analysis of variance (ANOVA) followed by Turkey’s honestly significant difference (HSD) test. Differences were considered significant at *p* < 0.05, with different letters indicating significant differences.

### 4.11. Subcellular Localization

Full-length coding sequences of *LbabZIP15*, *LbabZIP21*, *LbabZIP31*, *LbabZIP34*, *LbabZIP49*, and *LbabZIP65* (excluding stop codons) were cloned into the pCAMBIA2300-GFP vector. Recombinant constructs (pCAMBIA2300-*LbabZIP15/21/31/34/49/65*-GFP) were introduced into *A. tumefaciens* strain GV3101 via the heat shock method. Four-week-old *N. benthamiana* seedlings were used for transient expression via agrobacterium-mediated infiltration. Infiltrated leaves were kept in the dark for 12 h, and fluorescence signals were observed 72 h post-injection using a laser confocal microscope (Zeiss, Oberkochen, Germany). The pCAMBIA2300-GFP empty vector served as a negative control. Primer sequences are listed in Supplementary Table S10.

### 4.12. Transactivation Assays

The coding sequences (CDS) of *LbabZIP21/40/49/65* were cloned into the pGBKT7 vector to generate recombinant plastids (pGBKT7-*LbabZIP21/40/49/65*). Recombinant plasmids, along with the positive control (pGBKT7-53 + pGADT7-RecT) and the negative control (empty vector pGBKT7), were transformed into yeast strain AH109. Transformed yeast cells were grown on SD/-Leu/-Trp medium and transferred to SD/-Ade/-His/-Leu/-Trp medium to assess transactivation activity. Plates were incubated at 28 °C for three days, and transactivation activity was evaluated based on yeast cell growth. Primer sequences are listed in Supplemental Table S11.

## 5. Conclusions

This study provides the first genome-wide analysis of the *bZIP* gene family in wolfberry. A total of sixty-six wolfberry *bZIP* genes were identified, cis-acting element prediction was performed, and analyses of phylogenetic relationship, gene structure, motif composition, chromosomal distribution, collinearity, and expression patterns were performed. Phylogenetic analysis of *bZIP* genes from wolfberry, tomato, pepper, Arabidopsis, and rice revealed both conserved and divergent evolutionary patterns, providing instructive information for further investigation on the function of *LbaZIP* genes. Furthermore, study of expression patterns in diverse tissues revealed that *LbabZIP* played an important role in wolfberry growth and development. In addition, we analyzed the expression of *LbabZIP* genes at different fruit development stages and identified some candidate genes involved in the growth and development of wolfberry fruits. Our findings significantly advance understanding of bZIP-mediated regulation in wolfberry, providing both a framework for future functional studies of individual *LbabZIP* genes and valuable targets for molecular breeding of improved fruit quality traits.

## Data Availability

The wolfberry genome datasets used during the current study are available in NCBI database (https://www.ncbi.nlm.nih.gov/bioproject/PRJNA640228).The RNA-seq data used in this study were downloaded from the NCBI Database (PRJNA845109).

## Supplementary Materials

The following supporting information can be downloaded at https://www.mdpi.com/article/10.3390/ijms26104665/s1.
